# The Impact of Zinc Supplementation on Critically Ill Patients With Acute Kidney Injury: A Propensity Score Matching Analysis

**DOI:** 10.3389/fnut.2022.894572

**Published:** 2022-06-13

**Authors:** Wenkai Xia, Chenyu Li, Danyang Zhao, Lingyu Xu, Meisi Kuang, Xiajuan Yao, Hong Hu

**Affiliations:** ^1^Department of Nephrology, The Jiangyin People's Hospital Affiliated to Nantong University, Jiangyin, China; ^2^Nephrologisches Zentrum, Medizinische Klinik und Poliklinik IV, Klinikum der Universität München, Ludwig-Maximilians-University Munich, Munich, Germany; ^3^Department of Nephrology, The Affiliated Hospital of Qingdao University, Qingdao, China

**Keywords:** critical ill, acute kidney injury, zinc supplementation, sepsis, intensive care unit

## Abstract

**Background:**

Zinc is an essential trace element involved in multiple metabolic processes. Acute kidney injury (AKI) is associated with low plasma zinc, but outcomes with zinc supplementation in critically ill patients with AKI remain unknown. Our objective was to investigate the effectiveness of zinc supplementation in this patient population.

**Methods:**

Critically ill patients with AKI were identified from the Medical Informative Mart for Intensive Care IV database. Prosperity score matching (PSM) was applied to match patients receiving zinc treatment to those without zinc treatment. The association between zinc sulfate use and in-hospital mortality and 30-day mortality, need for renal replacement therapy (RRT), and length of stay was determined by logistic regression and Cox proportional hazards modeling.

**Results:**

A total of 9,811 AKI patients were included in the study. PSM yielded 222 pairs of patients who received zinc treatment and those who did not. Zinc supplementation was associated with reduced in-hospital mortality (HR = 0.48 (95% CI: 0.28, 0.83) *P* = 0.009) and 30-day mortality (HR = 0.51 (95% CI, 0.30, 0.86) *P* = 0.012). In the subgroup analysis, zinc use was associated with reduced in-hospital mortality in patients with stage 1 AKI and those with sepsis.

**Conclusions:**

Zinc supplementation was associated with improved survival in critically ill patients with AKI. The supplementation was especially effective in those with stage 1 AKI and sepsis. These results need to be verified in randomized controlled trials.

## Introduction

Acute kidney injury (AKI) is common in critically ill patients admitted to the intensive care unit (ICU), and its prevalence has risen as high as 50% in recent decades (1). To date, no specific treatment strategies have definitively improved outcomes in these patients. Current therapeutic approaches for critically ill patients with AKI revolve around volume status control (2), hemodynamic management (3, 4), renal replacement therapy (RRT) (5) and avoiding nephrotoxic drugs. However, whether this population benefits from these treatment options remains controversial (6). Fluid and electrolyte management, metabolic stabilization, and nutritional supplements including diuretics, sodium bicarbonate, ondansetron, and thiamine also have been investigated in critically ill patients with AKI (7–9).

Zinc is an essential micronutrient involved in numerous metabolic processes. Its deficiency results in immune dysfunction and infection, and zinc is viewed as fundamentally important in critical illness (10–12). Low plasma zinc has been observed in patients with AKI regardless of RRT treatment (13), and zinc has shown potential antioxidant activity in renal ischemia reperfusion injury (14). The benefit of zinc supplementation for the immune response has been demonstrated in both adults and critically ill children (15, 16). However, little evidence is available regarding the impact of zinc treatment on critically ill patients with AKI. In this propensity score matching (PSM) study, our objective was to investigate the efficacy of zinc supplementation in critically ill patients with AKI. We hypothesized that zinc supplementation as adjunctive therapy would be associated with improved survival in this patient population.

## Methods

### Database

The study data were extracted from the publicly available Multiparameter Intelligent Monitoring in Intensive Care IV (MIMIC IV, version 1.0) database. These data cover more than 50,000 critically ill patients admitted to the Beth Israel Deaconess Medical Center during 2008 to 2019 (17). Access to the database was approved by the institutional review boards of the Massachusetts Institute of Technology (No. 0403000206) and the Beth Israel Deaconess Medical Center (2001-P-001699/14). One author (LX) had access to this database (Certification Number 37851920) and retrieved the data on October 15, 2021. Given that all data were anonymous, informed consent was waived. All procedures were performed in compliance with relevant guidelines and regulations.

### Population Selection Criteria

Adults (≥18 years) who were admitted to ICU for more than 48 h with confirmed AKI according to the Kidney Disease: Improving Global Outcomes (KDIGO) criteria were eligible for inclusion. The KDIGO criteria (18) were as follows: an increase in serum creatinine by 50% from baseline within the previous 7 days, increase in serum creatinine by 0.3 mg/dl within the last 48 h, or oliguria (urine output <0.5 ml/kg/h) for 6 h or more. Baseline serum creatinine level was considered to be the minimum serum creatinine within 7 days prior to admission. The first serum creatinine measured at ICU admission was used as the baseline value when preadmission serum creatinine was not available. Serum creatinine during the first 48 h after ICU admission was used to define AKI stage. Patients were excluded if they were discharged or died within 48 h after ICU admission. For patients with multiple ICU admissions, only the first admission was included.

### Endpoints

The objective of the current study was to investigate the efficiency of zinc sulfate as adjunctive therapy in critically ill patients with AKI. The primary endpoint was in-hospital mortality, defined as survival status at hospital discharge. Secondary outcomes were 30-day mortality, need for RRT, ICU length of stay (LOS), and hospital LOS.

### Data Collection and Definitions

Data for each patient within 24 h of ICU admission were collected from the MIMIC IV database and managed using Structured Query Language (i.e., SQL) with Navicat Premium (version 9.6). The extracted information included age, sex, ethnicity, admission type, platelets, red blood cell (RBC) count, hemoglobin, white blood cell count, serum creatinine, anion gap, international normalized ratio (INR), activated partial thromboplastin time (APTT), glucose, simplified acute physiology score II (SAPS II), estimated glomerular filtration rate (eGFR), AKI stage, mean arterial pressure (MAP), RRT, mechanical ventilation, and use of vasopressors, antibiotics, or anticoagulants. In addition, the following comorbidities were included, all collected and defined according to the Implementation of the International Statistical Classification of Disease and Related Health Problems, 10^th^ Revision: chronic kidney disease (CKD), hypertension, diabetes, heart failure, cancer, coronary artery disease, chronic obstructive pulmonary disease (COPD), acute respiratory distress syndrome (ARDS), stroke chronic liver disease, and sepsis (19). Zinc supplementation was defined as an oral intake of 50 mg/d zinc element provided by zinc sulfate tablets. Sepsis was defined as a recorded or suspected infection plus a Sequential Organ Failure Assessment score ≥2 based on the diagnostic criteria of the International Consensus Definitions for Sepsis and Septic Shock (20). CKD was defined as structural or functional injury for more than 3 months. Missing data variables in the MIMIC IV database were common, and in the present study, all variables had <5% missing values (Supplementary Table S1). Components were removed from the study if the proportion of missing values reached 20%, and removed factors included C-reactive protein, albumin, triglycerides, cholesterol, and serum lactate.

### Statistical Analysis

Continuous variables in the current study are expressed as mean ± standard deviation or median with interquartile range and were compared using the student's *t*-test or the Mann–Whitney *U* test as appropriate. Categorical variables are presented as numbers and percentages and were compared using the Chi-square or Fisher's exact test.

PSM was performed to match patients who received zinc sulfate supplementation over the recommended dietary allowance to those who did not. Patients were matched in a 1:1 greedy nearest neighbor algorithm with a caliper width of 0.2. We generated the propensity score according to the following variables: age, sex, ethnicity, admission type, platelet, RBC count, hemoglobin, white blood cell count, serum creatinine, anion gap, INR, APTT, glucose, MAP, eGFR, vasopressor use, antibiotic use, anticoagulant use, AKI stage, SAPS II at ICU admission, CKD, diabetes, hypertension, heart failure, COPD, ARDS, cancer, chronic liver disease, coronary artery disease, stroke, and sepsis. Standardized mean difference was applied before and after matching to evaluate the efficiency of PSM in reducing differences between the two groups. Finally, 222 matched pairs were established for further analysis.

Multivariable Cox proportional hazards regression analysis was used to estimate the relationship between zinc sulfate administration and mortality, with adjustments for confounding variables based on *P* < 0.05 in univariate analysis and potential confounders judged by clinical expertise. Linear regression was performed to evaluate the association between use of zinc sulfate and LOS. Data are given as hazard ratios (HRs) with 95% confidence intervals (CIs).

Stratification analysis was performed to explore whether the association between zinc sulfate administration and in-hospital mortality differed across various subgroups classified by AKI stage, CKD, diabetes, hypertension, heart failure, chronic liver disease, ARDS, cancer, coronary artery disease, and sepsis in the population after PSM matching.

All statistical analyses were performed using SPSS 21.0 (SPSS Inc., IBM, USA) and R 3.5.3. A *P* < 0.05 was considered to be statistically significant.

## Results

### Study Population

**Figure 1:**
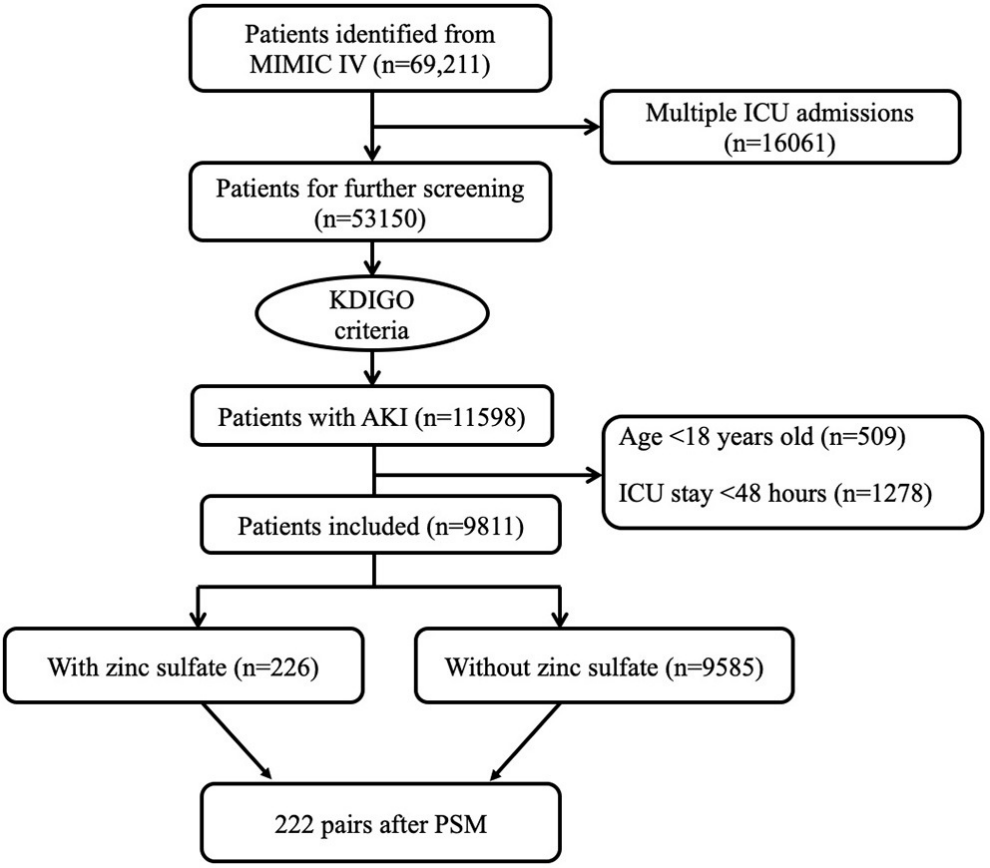
Flowchart of patient selection. ICU, intensive care unit; MIMIC IV, Multiparameter Intelligent Monitoring in Intensive Care Database IV; PSM, propensity score matching.

A total of 115,985 critically ill patients with AKI were admitted to the ICU during the study period. According to the exclusion criteria, 9,811 eligible patients were fully enrolled. Of these, 226 patients were exposed to zinc sulfate once daily within 48 h after ICU admission, whereas 9,585 patients did not receive zinc sulfate therapy (Figure 1).

Before PSM, there were significant differences in ethnicity and admission type between the two groups. The baseline RBC count, hemoglobin, and eGFR were higher in the non-zinc group when compared to the zinc group. Conversely, patients in the zinc group had a higher INR. Vasopressor use and anticoagulant use were more common in the zinc group. Patients with CKD were more likely to be given zinc sulfate (Table 1).

**Table 1:** Baseline characteristics of groups before propensity score matching analysis.

**Variables**	**Non-zinc group** **(*n* = 9,585)**	**Zinc group** **(*n* = 226)**	***P***	**SMD**
Age	67 ± 15	65 ± 14	0.090	0.136
Sex, male, *n* (%)	5,743 (59.9)	125 (55.3)	0.163	0.075
**Ethnicity**, ***n*** **(%)**			0.001	0.178
White	6,185 (64.5)	156 (69.0)		
Black	870 (9.1)	32 (14.2)		
Other	2,530 (26.4)	38 (16.8)		
**Admission type**, ***n*** **(%)**			0.004	0.177
Observation	999 (10.4)	37 (16.4)		
Elective	494 (5.2)	7 (3.1)		
Emergency	5,694 (59.4)	140 (61.9)		
Urgent	2,398 (25.0)	42 (18.6)		
**Laboratory parameters**				
Platelets, 10^9^/L	195.4 ± 110.9	200.0 ± 126.5	0.542	0.036
RBC count, 10^9^/L	3.4 ± 0.8	3.2 ± 0.7	<0.001	0.291
Hemoglobin, g/dl	10.3 ± 2.3	9.6 ± 1.9	<0.001	0.384
WBC count, 10^9^/L	13.2 ± 9.7	13.4 ± 7.3	0.762	0.013
Serum creatinine	1.9 ± 2.1	2.2 ± 2.2	0.051	0.145
Anion gap, mmol/L	15.7 ± 5.1	15.8 ± 5.0	0.699	0.035
APTT, seconds	41.6 ± 27.1	42.3 ± 27.0	0.692	0.029
INR	1.6 ± 1.0	1.7 ± 1.1	0.024	0.134
Glucose, mg/dl	153.9 ± 86.4	148.6 ± 66.7	0.318	0.078
**Co-morbidities**, ***n*** **(%)**				
CKD	1,791 (18.7)	62 (27.4)	0.001	0.199
Diabetes	2,042 (21.3)	58 (25.7)	0.114	0.093
Heart failure	2,041 (21.3)	55 (24.3)	0.270	0.070
Hypertension	2,398 (25.0)	44 (19.5)	0.057	0.155
Chronic liver disease	558 (5.8)	17 (7.5)	0.282	0.067
COPD	170 (1.8)	3 (1.3)	0.801	0.091
ARDS	2,003 (20.9)	45 (19.9)	0.719	0.039
Cancer	1,042 (10.9)	19 (8.42)	0.238	0.083
Coronary artery disease	2,085 (21.8)	42 (18.6)	0.253	0.100
Stroke	275 (2.9)	5 (2.2)	0.558	0.043
Sepsis	1,271 (13.3)	40 (17.7)	0.053	0.122
MAP, mmHg	82.4 ± 19.2	80.4 ± 18.3	0.116	0.106
eGFR, ml/min/1.73 m^2^	58.1 ± 34.6	52.6 ± 36.0	0.018	0.160
Mechanical ventilation, *n* (%)	6,171 (64.4)	150 (66.4)	0.537	0.040
Vasopressor use, *n* (%)	3,663 (38.2)	102 (45.1)	0.035	0.147
Anticoagulant use, *n* (%)	785 (8.2)	32 (14.2)	0.001	0.174
Antibiotic use, *n* (%)	7,432 (77.5)	186 (82.3)	0.089	0.118
**AKI stage**, ***n*** **(%)**			0.117	0.053
1	7,848 (81.9)	183 (81.0)		
2	432 (4.5)	5 (2.2)		
3	1,305 (13.6)	38 (16.8)		
**Scoring systems**				
SAPSII score	43.2 ± 14.9	44.9 ± 15.3	0.105	0.177

*AKI, acute kidney injury; ARDS, acute respiratory distress syndrome; APTT, activated partial thromboplastin time; COPD, chronic obstructive pulmonary disease; eGFR, estimated glomerular filtration rate; INR, international normalized ratio; MAP, mean arterial pressure; RBC, red blood cell; SAPSII, Simplified Acute Physiology Score II; SMD, standardized mean difference; WBC, white blood cell*.

### Association Between Zinc Sulfate and Clinical Outcomes

A multivariable Cox proportional hazard model was run for clinical outcomes between the two groups. In the pre-matched cohort, zinc sulfate use was associated with reduced in-hospital mortality [HR = 0.59 (95% CI: 0.40, 0.89) *P* = 0.011] and 30-day mortality [HR = 0.56 (95% CI: 0.32, 0.85) *P* = 0.009] after adjustment for confounding factors associated with mortality (Supplementary Table S2). A logistic regression model was used to estimate the impact of zinc sulfate on RRT, showing that zinc sulfate was associated with increased need for RRT [HR = 1.57 (95% CI: 1.07, 2.32) *P* = 0.023]. Furthermore, zinc sulfate use was associated with longer ICU LOS [HR = 1.80 (95% CI: 1.34, 2.42) *P* < 0.001] and hospital LOS [HR = 1.35 (95% CI: 1.21, 2.32) *P* < 0.001] (Table 2).

**Table 2:** Association of zinc use with clinical outcome in critically ill patients with AKI.

	**Non-zinc group**	**Zinc group**	***P***	**Unadjusted HR (95% CI)**	***P***	**Adjusted HR (95% CI)**	***P***
Pre-matched cohort	*n* = 9,585	*n* = 226					
**Primary outcome**							
In-hospital mortality, *n* (%)^#^	2,156 (22.5)	36 (15.9)	0.019	0.49 (0.36, 0.69)	<0.001	0.59 (0.40, 0.89)	0.011
**Secondary outcomes**							
RRT^$^	1,657 (17.3)	60 (26.5)	<0.001	1.73 (1.28, 2.33)	<0.001	1.57 (1.07, 2.32)	0.023
30-day mortality, *n* (%)^#^	2,317 (24.2)	39 (17.3)	0.016	0.65 (0.46, 0.93)	0.017	0.56 (0.32, 0.85)	0.009
Length of ICU stay*	5.9 ± 5.2	7.8 ± 6.4	<0.001	1.79 (1.36, 2.35)	<0.001	1.80 (1.34, 2.42)	<0.001
Length of hospital stay*	11.7 ± 6.9	15.8 ± 7.2	<0.001	3.17 (2.33, 4.31)	<0.001	1.35 (1.21, 2.31)	<0.001
Post-matched cohort	*n* = 222	*n* = 222					
**Primary outcome**							
In-hospital mortality, *n* (%)^#^	55 (24.8)	36 (16.2)	0.022	0.43 (0.28, 0.66)	<0.001	0.48 (0.28, 0.83)	0.009
**Secondary outcomes**							
RRT^$^	59 (26.6)	60 (26.4)	0.915	1.02 (0.67, 1.56)	0.915	1.31 (0.72, 2.39)	0.371
30-day mortality, *n* (%)^#^	58 (26.1)	39 (17.6)	0.029	0.60 (0.38, 0.95)	0.030	0.51 (0.30, 0.86)	0.012
Length of ICU stay*	6.4 ± 5.7	7.8 ± 6.4	0.018	0.81 (0.56, 1.17)	0.255	0.86 (0.58, 1.27)	0.444
Length of hospital stay*	12.2 ± 6.9	15.9 ± 7.2	<0.001	1.02 (0.70, 1.48)	0.924	0.97 (0.65, 1.45)	0.895

*AKI, acute kidney injury; CI, confidence interval; HR, hazard ratio; ICU, intensive care unit; RRT, renal replacement therapy.*

*#Cox regression was used for estimating the impact of zinc sulfate use on mortality outcomes, with adjustment for confounding variables selected based on P <0.05 in univariate analysis.*

*$Logistic regression analysis was used to evaluate the association of zinc sulfate and RRT.*

*A generalized linear model was used to calculate beta coefficients (estimates) and P-values.

After PSM, 222 patients who received zinc sulfate were matched to 222 patients who did not. The baseline characteristics and comorbidities were well balanced between the two groups, and the standardized differences of the means were provided (Supplementary Table S3). Among the 222 propensity-matched pairs, zinc sulfate use was associated with reduced in-hospital mortality [HR = 0.48 (95% CI: 0.28, 0.83) *P* = 0.009] and 30-day mortality [HR = 0.51 (95% CI: 0.30, 0.86) *P* = 0.012]. However, zinc sulfate use was not associated with the application of RRT [HR = 1.31 (95% CI: 0.72, 2.39) *P* = 0.371], ICU LOS [HR = 0.86 (95% CI: 0.58, 1.27) *P* = 0.444], or hospital LOS [HR = 0.97 (95% CI: 0.65, 1.45) *P* = 0.895] (Table 2).

### Subgroup Analysis

According to the KDIGO criteria, zinc sulfate use was associated with reduced in-hospital mortality in patients with stage 1 AKI but not in those with stage 2 or 3 AKI. Of interest, the improved in-hospital outcome was observed in patients with sepsis in the ICU [HR = 0.25 (95% CI: 0.11, 0.57) *P* = 0.001]. When the analysis was restricted to patients with diabetes, heart failure, or hypertension, zinc sulfate administration was not associated with hospital outcome. Similar analyses in other groups were not significant (Table 3).

**Table 3:** The association between zinc sulfate therapy and in-hospital mortality in subgroups.

**Subgroup**	***N***	**HR**	**95% CI**	***P***	***P* for interaction**
**AKI stages**					0.006
1	358	0.39	0.24, 0.64	<0.001	
2–3	86	0.75	0.34, 1.65	0.795	
**CKD**					0.083
No	321	0.45	0.28, 0.73	0.001	
Yes	123	0.16	0.06, 0.45	<0.001	
**Diabetes**					0.010
No	321	0.45	0.28, 0.73	0.001	
Yes	123	0.16	0.06, 0.45	<0.001	
**Heart failure**					0.001
No	331	0.44	0.27, 0.70	0.001	
Yes	113	0.28	0.10, 0.78	0.015	
**Hypertension**					0.019
No	354	0.47	0.30, 0.75	0.002	
Yes	90	0.05	0.01, 0.24	<0.001	
**Chronic liver disease**					0.292
No	410	0.49	0.31, 0.77	0.002	
Yes	34	0.33	0.07, 1.68	0.182	
**ARDS**					0.098
No	356	0.52	0.31, 0.87	0.012	
Yes	88	0.23	0.09, 0.57	0.001	
**Cancer**					0.340
No	401	0.52	0.33, 0.83	0.006	
Yes	43	0.71	0.21, 1.85	0.058	
**Coronary artery disease**					0.454
No	359	0.40	0.25, 0.64	<0.001	
Yes	85	0.67	0.14, 1.81	0.449	
**Sepsis**					0.018
No	355	0.63	0.37, 1.06	0.081	
Yes	89	0.25	0.11, 0.57	0.001	

*AKI, acute kidney injury; ARDS, acute respiratory distress syndrome; CI, confidence interval; CKD, chronic kidney disease; HR, hazard ratio*.

## Discussion

The present study demonstrated an association of zinc supplementation with reduced in-hospital mortality and 30-day mortality in critically ill patients with AKI, even after adjustment for major covariates. Results of the subgroup analysis suggested that zinc treatment might have a beneficial impact on patients with stage 1 AKI according to KDIGO criteria.

There are several possible mechanisms by which zinc treatment could exert beneficial effects on critically ill patients with AKI. Zinc is required for both the innate and adaptive immune systems. Zinc deficiency induces a cumulative loss of B cell and T cell maturation, which subsequently results in lymphopenia and impaired natural killer (NK) cell function (10, 21). It has been proposed that zinc administration could restore lymphocyte production and NK cell activity (22). Zinc also has been suggested to regulate metallothionein, which has a role in free radical scavenging and the inflammatory response (23). Additionally, abnormal elevation of blood glucose has been recognized as an indicator of poor prognosis in critically ill patients (24, 25), and the presence of zinc is essential for insulin secretion and glucose homeostasis (26, 27). Of interest, in animal models of bowel anastomosis, zinc supplementation has enhanced wound healing in cutaneous and gastrointestinal wounds (28). Thus, zinc use could be an indispensable medical option in patients with surgical and burn trauma.

Mild to moderate zinc deficiency is often found during the early stages of patient care after admission to the ICU because of increased metabolic rate, inadequate nutritional intake, and ongoing feeding difficulties. In most cases, plasma zinc concentration seems to reflect poor nutrition. However, in patients with an inflammatory response, rapid declines in zinc are partially the result of its redistribution into the cellular compartment (29, 30). Consequently, measurement of plasma zinc, especially in critically ill patients with AKI, can be uninformative and may be misleading. Of note, the decision to use zinc supplementation for patients, particularly in the ICU, solely depends on physician clinical judgment according to indirect clues, such as clinical characteristics (i.e., impaired wound healing, acrodermatitis enteropathica), poor nutrition, and related lab values (i.e., serum alkaline phosphatase, a zinc-dependent metalloenzyme) (31).

In our study, we found an association in the unmatched analysis between zinc supplementation and longer ICU and hospital LOS. There were differences in mortality rates between the two groups, and some patients who died early in the non-zinc group would have had shorter ICU or hospital LOS. We also found, however, that zinc use was not independently associated with ICU or hospital stay in the PSM analysis. A systematic review of four randomized controlled trials (RCTs) of zinc supplementation in non-critically ill patients showed no effect on ICU or hospital LOS or duration of mechanical ventilation (32). Most such studies have included patients with mild and moderate disease, and further investigations are needed to confirm any benefits of zinc use, whether for all critically ill patients with AKI or exclusively for those with zinc deficiency.

Subgroup analysis of various AKI stages indicated that the beneficial effect of zinc supplementation on mortality was especially observed in patients with stage 1 AKI. However, stage 2-3 AKI patients represented more severe clinical cases, and it is possible that this population may not benefit from zinc supplementation. A recent study of critically ill patients in the ICU with COVID-19, however, showed an association of oral zinc supplementation with reduced AKI incidence (33). An exact mechanism for a renal protective effect of zinc has not been identified, but zinc may mitigate AKI risk through antioxidant action (14). The potential of zinc in renal protection in critically ill patients warrants further study.

Our results additionally show that zinc supplementation was independently associated with reduced mortality in patients with sepsis. This finding is consistent with previous studies showing that zinc supplementation improved survival rates in murine models of sepsis (34, 35). However, data conflict on the association of zinc use with clinical outcomes in the context of sepsis in humans. Some relevant RCTs have shown a beneficial effect of zinc use in reducing mortality rate and improving neurological development in neonates (36, 37). In contrast, Newton et al. found that the use of zinc had no notable impact on survival rate and hospital stay in neonatal sepsis (38). Of note, in another study, parenteral zinc administration was associated with an exaggerated acute phase response (39) and potentially interfered with nutritional status. A recent meta-analysis comparing outcomes for hospitalized patients receiving zinc with a control intervention (15) showed that zinc supplementation significantly reduced mortality in sepsis, in agreement with our current finding of survival benefit. Most of these previous studies involved neonatal sepsis.

Here, using PSM, we provide initial results supporting zinc supplementation in critically ill patients with AKI. Currently, evidence is limited for zinc as an adjunctive treatment in this patient population. Some studies have evaluated zinc supplementation in critically ill patients with COVID-19, and an ongoing RCT is assessing intravenous zinc in this patient population (40).

Several potential limitations in the current study should be acknowledged. First, because of the single-center retrospective design, we cannot rule out unknown confounder effects. A multi-center reearch could provide a more robust and representative evidence. Second, zinc was administered by the enteral route in our study, and many factors could lead to poor absorption of zinc in critically ill patients, such as malabsorption syndrome and inflammatory diseases of the bowel. Although plasma zinc is not routinely measured, it was thought to be useful as a diagnostic marker for evaluating the severity of sepsis. A prospective cohort trial monitoring zinc levels after zinc therapy *via* parenteral route would enable us to better investigate the association between zinc supplementation and prognosis in these population. Third, in the absence of evidence-based medical guidelines, zinc administration decisions were left to the discretion of the clinicians, which might be a source of bias. More evidence was warranted to further clarify the beneficial effect of zinc supplementation in ICU patients. Forth, the number of patients in the stage 2-3 AKI group was relatively small, which may have resulted in selection bias and affected statistical significance. A longitudinal study with longer follow-up as well as a large sample size should be considered and studied in the future. Finally, given the ongoing pandemic, it cannot be overstated that using supplements in critical cases risks side effects for ICU patients. Further prospective studies should be considered. Thus, these findings should not be used to guide clinical practice and rather can serve to highlight the need for further investigation into the potential benefits of zinc supplementation in critically ill patients with AKI.

## Conclusion

Zinc supplementation is associated with improved outcomes in critically ill patients with stage 1 AKI and sepsis. Well-designed prospective studies are needed to confirm these findings.

## Data Availability Statement

The clinical data used to support the findings of this study was supplied by Monitoring in Intensive Care Database III version 1.4 (MIMIC-III v.1.4). Although the database is publicly and freely available, researchers must complete the National Institutes of Health's web-based course known as Protecting Human Research Participants to apply for permission to access the database. The datasets used and analyzed during the current study are available from the corresponding author on reasonable request.

## Ethics Statement

The studies involving human participants were reviewed and approved by MIMIC III database used in the present study, the Institutional Review Boards (IRB) of the Massachusetts Institute of Technology and does not contain protected health information. The Ethics Committee waived the requirement of written informed consent for participation.

## Author Contributions

WX designed the study and drafted the manuscript. CL and LX analyzed the data and extracted database. DZ contributed to scientific discussion and data interpretations. MK contributed to scientific discussion. XY prepared the tables. HH supervised the study and reviewed the manuscript. All authors contributed to the article and approved the submitted version.

## Conflict of Interest

The authors declare that the research was conducted in the absence of any commercial or financial relationships that could be construed as a potential conflict of interest.

## Publisher's Note

All claims expressed in this article are solely those of the authors and do not necessarily represent those of their affiliated organizations, or those of the publisher, the editors and the reviewers. Any product that may be evaluated in this article, or claim that may be made by its manufacturer, is not guaranteed or endorsed by the publisher.

## References

[1] Hoste EA Bagshaw SM Bellomo R Cely CM Colman R Cruz DN. Epidemiology of acute kidney injury in critically ill patients: the multinational AKI-EPI study. Intensive Care Med. (2015) 41:1411–23. 10.1007/s00134-015-3934-7 26162677

[2] Ostermann M Liu K Kashani K. Fluid management in acute kidney injury. Chest. (2019) 156:594–603. 10.1016/j.chest.2019.04.004 31002784

[3] Asano E. High-frequency oscillations are under your control. Don't chase all of them. Clin Neurophysiol. (2017) 128:841–2. 10.1016/j.clinph.2017.02.003 28283356 PMC5567820

[4] Brar SS Aharonian V Mansukhani P Moore N Shen AY Jorgensen M. Haemodynamic-guided fluid administration for the prevention of contrast-induced acute kidney injury: the POSEIDON randomised controlled trial. Lancet. (2014) 383:1814–23. 10.1016/S0140-6736(14)60689-9 24856027

[5] Tolwani A. Continuous renal-replacement therapy for acute kidney injury. N Engl J Med. (2012) 367:2505–14. 10.1056/NEJMct1206045 23268665

[6] Kellum JA Romagnani P Ashuntantang G Ronco C Zarbock A Anders HJ. Acute kidney injury. Nat Rev Dis Primers. (2021) 7:52. 10.1038/s41572-021-00284-z 34267223

[7] Zhao GJ Xu C Ying JC Lu WB Hong GL Li MF. Association between furosemide administration and outcomes in critically ill patients with acute kidney injury. Crit Care. (2020) 24:75. 10.1186/s13054-020-2798-6 32131879 PMC7057586

[8] Timal RJ Kooiman J Sijpkens YWJ de Vries JPM Verberk-Jonkers I Brulez HFH. Effect of no prehydration vs. sodium bicarbonate prehydration prior to contrast-enhanced computed tomography in the prevention of postcontrast acute kidney injury in adults with chronic kidney disease: the kompas randomized clinical trial. JAMA Intern Med. (2020) 180:533–41. 10.1001/jamainternmed.2019.7428 32065601 PMC7042862

[9] Li X Luan H Zhang H Li C Bu Q Zhou B. Associations between early thiamine administration and clinical outcomes in critically ill patients with acute kidney injury. Br J Nutr. (2021). 10.1017/S0007114521003111 [Epub ahead of print]. 34392848

[10] Fraker PJ King LE. Reprogramming of the immune system during zinc deficiency. Annu Rev Nutr. (2004) 24:277–98. 10.1146/annurev.nutr.24.012003.132454 15189122

[11] Skrajnowska D Bobrowska-Korczak B. Role of zinc in immune system and anti-cancer defense mechanisms. Nutrients. (2019) 11:2273. 10.3390/nu11102273 31546724 PMC6835436

[12] Bonaventura P Benedetti G Albarede F Miossec P. Zinc and its role in immunity and inflammation. Autoimmun Rev. (2015) 14:277–85. 10.1016/j.autrev.2014.11.008 25462582

[13] Ostermann M Summers J Lei K Card D Harrington DJ Sherwood R. Micronutrients in critically ill patients with severe acute kidney injury—a prospective study. Sci Rep. (2020) 10:1505. 10.1038/s41598-020-58115-2 32001725 PMC6992767

[14] Akbari G. Role of zinc supplementation on ischemia/reperfusion injury in various organs. Biol Trace Elem Res. (2020) 196:1–9. 10.1007/s12011-019-01892-3 31828721

[15] Tang Z Wei Z Wen F Wu Y. Efficacy of zinc supplementation for neonatal sepsis: a systematic review and meta-analysis. J Matern Fetal Neonatal Med. (2019) 32:1213–8. 10.1080/14767058.2017.1402001 29103346

[16] Besecker BY Exline MC Hollyfield J Phillips G Disilvestro RA Wewers MD. A comparison of zinc metabolism, inflammation, and disease severity in critically ill infected and noninfected adults early after intensive care unit admission. Am J Clin Nutr. (2011) 93:1356–64. 10.3945/ajcn.110.008417 21525204 PMC3095505

[17] Johnson AE Pollard TJ Shen L Lehman LW Feng M Ghassemi M. MIMIC-III, a freely accessible critical care database. Sci Data. (2016) 3:160035. 10.1038/sdata.2016.35 27219127 PMC4878278

[18] Andrassy KM. Comments on KDIGO 2012 clinical practice guideline for the evaluation and management of chronic kidney disease. Kidney Int. (2013) 84:622–3. 10.1038/ki.2013.243 23989362

[19] Quan H Sundararajan V Halfon P Fong A Burnand B Luthi JC. Coding algorithms for defining comorbidities in ICD-9-CM and ICD-10 administrative data. Med Care. (2005) 43:1130–9. 10.1097/01.mlr.0000182534.19832.83 16224307

[20] Singer M Deutschman CS Seymour CW Shankar-Hari M Annane D Bauer M. The third international consensus definitions for sepsis and septic shock (sepsis-3). JAMA. (2016) 315:801–10. 10.1001/jama.2016.0287 26903338 PMC4968574

[21] Rink L Kirchner H. Zinc-altered immune function and cytokine production. J Nutr. (2000) 130:1407S−11. 10.1093/jn/130.5.1407S 10801952

[22] Shankar AH Prasad AS. Zinc and immune function: the biological basis of altered resistance to infection. Am J Clin Nutr. (1998) 68:447S−63. 10.1093/ajcn/68.2.447S 9701160

[23] Choi S Liu X Pan Z. Zinc deficiency and cellular oxidative stress: prognostic implications in cardiovascular diseases. Acta Pharmacol Sin. (2018) 39:1120–32. 10.1038/aps.2018.25 29926844 PMC6289396

[24] Falciglia M Freyberg RW Almenoff PL D'Alessio DA Render ML. Hyperglycemia-related mortality in critically ill patients varies with admission diagnosis. Crit Care Med. (2009) 37:3001–9. 10.1097/CCM.0b013e3181b083f7 19661802 PMC2905804

[25] Godinjak A Iglica A Burekovic A Jusufovic S Ajanovic A Tancica I. Hyperglycemia in critically ill patients: management and prognosis. Med Arch. (2015) 69:157–60. 10.5455/medarh.2015.69.157-160 26261382 PMC4500381

[26] Fernandez-Cao JC Warthon-Medina M Arija HMVV Doepking C Serra-Majem L Lowe NM. Zinc intake and status and risk of type 2 diabetes mellitus: a systematic review and meta-analysis. Nutrients. (2019) 11:1027. 10.3390/nu11051027 31071930 PMC6567047

[27] Bjorklund G Dadar M Pivina L Dosa MD Semenova Y Aaseth J. The role of zinc and copper in insulin resistance and diabetes mellitus. Curr Med Chem. (2020) 27:6643–57. 10.2174/0929867326666190902122155 31475889

[28] Lansdown AB Mirastschijski U Stubbs N Scanlon E Agren MS. Zinc in wound healing: theoretical, experimental, and clinical aspects. Wound Repair Regen. (2007) 15:2–16. 10.1111/j.1524-475X.2006.00179.x 17244314

[29] Black RE. Zinc deficiency, infectious disease and mortality in the developing world. J Nutr. (2003) 133:1485S−9. 10.1093/jn/133.5.1485S 12730449

[30] Galloway P McMillan DC Sattar N. Effect of the inflammatory response on trace element and vitamin status. Ann Clin Biochem. (2000) 37:289–97. 10.1258/0004563001899429 10817241

[31] Ueda T Fujita G Yanagita T Kaise C Sato M. [Risk factors for mental health problems and complicated grief in bereaved families of motor vehicle accident victims]. Shinrigaku Kenkyu. (2017) 87:569–78. 10.4992/jjpsy.87.15028 29630292

[32] Derwand R Scholz M Zelenko V. COVID-19 outpatients: early risk-stratified treatment with zinc plus low-dose hydroxychloroquine and azithromycin: a retrospective case series study. Int J Antimicrob Agents. (2020) 56:106214. 10.1016/j.ijantimicag.2020.106214 33122096 PMC7587171

[33] Al Sulaiman K Aljuhani O Al Shaya AI Kharbosh A Kensara R Al Guwairy A. Evaluation of zinc sulfate as an adjunctive therapy in COVID-19 critically ill patients: a two center propensity-score matched study. Crit Care. (2021) 25:363. 10.1186/s13054-021-03785-1 34663411 PMC8522856

[34] Nowak JE Harmon K Caldwell CC Wong HR. Prophylactic zinc supplementation reduces bacterial load and improves survival in a murine model of sepsis. Pediatr Crit Care Med. (2012) 13:e323–9. 10.1097/PCC.0b013e31824fbd90 22760431 PMC3438373

[35] Knoell DL Julian MW Bao S Besecker B Macre JE Leikauf GD. Zinc deficiency increases organ damage and mortality in a murine model of polymicrobial sepsis. Crit Care Med. (2009) 37:1380–8. 10.1097/CCM.0b013e31819cefe4 19242332 PMC2905048

[36] Banupriya N Vishnu Bhat B Benet BD Sridhar MG Parija SC. Efficacy of zinc supplementation on serum calprotectin, inflammatory cytokines and outcome in neonatal sepsis - a randomized controlled trial. J Matern Fetal Neonatal Med. (2017) 30:1627–31. 10.1080/14767058.2016.1220524 27491377

[37] Banupriya N Bhat BV Benet BD Catherine C Sridhar MG Parija SC. Short term oral zinc supplementation among babies with neonatal sepsis for reducing mortality and improving outcome—a double-blind randomized controlled trial. Indian J Pediatr. (2018) 85:5–9. 10.1007/s12098-017-2444-8 28891027

[38] Newton B Bhat BV Dhas BB Mondal N Gopalakrishna SM. Effect of zinc supplementation on early outcome of neonatal sepsis—a randomized controlled trial. Indian J Pediatr. (2016) 83:289–93. 10.1007/s12098-015-1939-4 26616409

[39] Braunschweig CL Sowers M Kovacevich DS Hill GM August DA. Parenteral zinc supplementation in adult humans during the acute phase response increases the febrile response. J Nutr. (1997) 127:70–4. 10.1093/jn/127.1.70 9040547

[40] Perera M El Khoury J Chinni V Bolton D Qu L Johnson P. Randomised controlled trial for high-dose intravenous zinc as adjunctive therapy in SARS-CoV-2 (COVID-19) positive critically ill patients: trial protocol. BMJ Open. (2020) 10:e040580. 10.1136/bmjopen-2020-040580 33268419 PMC7712927

