# Supplementary material for: The Impact of Zinc Supplementation on Critically Ill Patients With Acute Kidney Injury: A Propensity Score Matching Analysis
Source: Front Nutr. 2022 Jun 13;9:894572. doi: 10.3389/fnut.2022.894572 (PMC9234667; doi:10.3389/fnut.2022.894572)
Supplement: Supplementary file 1 [file Data_Sheet_1.docx]

**Table S1. Missing proportions (%) for risk variables in the dataset**

| Variables | Missing, n (%) |
| --- | --- |
| Age | 0 (0) |
| Sex | 0 (0) |
| Admission type | 0 (0) |
| RRT | 0 (0) |
| Mechanical ventilation | 0 (0) |
| Use of zinc | 0 (0) |
| Vasopressor use | 0 (0) |
| Antibiotic use | 0 (0) |
| Anticoagulant use | 0 (0) |
| Platelet | 0 (0) |
| RBC count | 0 (0) |
| Hemoglobin | 0 (0) |
| WBC count | 0 (0) |
| Serum creatinine | 0 (0) |
| Anion gap | 0 (0) |
| APTT | 145 (1.3) |
| INR | 133 (1.2) |
| Glucose | 0 (0) |
| CKD | 0 (0) |
| Diabetes | 0 (0) |
| Heart failure | 0 (0) |
| Hypertension | 0 (0) |
| Chronic liver disease | 0 (0) |
| COPD | 0 (0) |
| ARDS | 0 (0) |
| Cancer | 0 (0) |
| Coronary artery disease | 0 (0) |
| Stroke | 0 (0) |
| Sepsis | 0 (0) |
| MAP | 2 (0.02) |
| eGFR | 0 (0) |
| AKI stage | 0 (0) |
| Hospital mortality | 0 (0) |
| 30-day mortality | 0 (0) |
| Length of hospital stay | 0 (0) |
| Length of ICU stay | 0 (0) |
| SAPSII | 0 (0) |

Abbreviation: AKI, acute kidney injury; ﻿APTT, activated partial thromboplastin time; ﻿ARDS, acute respiratory distress syndrome; COPD, chronic obstructive pulmonary disease; ﻿eGFR, estimated glomerular filtration rate; ICU, intensive care unit; ﻿INR, international normalized ratio; MAP, mean arterial pressure; RBC, red blood cell; ﻿RRT, renal replacement therapy; SAPSII, Simplified Acute Physiology Score II; WBC, white blood cell.

**Table S2. Potential risk variables for in-hospital mortality**

| Variables | HR | 95% CI | *P* |
| --- | --- | --- | --- |
| Age | 1.01 | 1.01, 1.02 | <0.001 |
| Sex, male | 1.11 | 1.02, 1.21 | 0.015 |
| Admission type | 0.97 | 0.75, 1.27 | 0.847 |
| Ethnicity, white | 1.08 | 1.03, 1.13 | 0.001 |
| RRT | 0.90 | 0.81, 1.01 | 0.062 |
| Mechanical ventilation | 1.05 | 0.94, 1.18 | 0.361 |
| Vasopressor use | 1.48 | 1.35, 1.63 | <0.001 |
| Antibiotic use | 0.86 | 0.77, 0.97 | 0.017 |
| Anticoagulant use | 0.72 | 0.60, 0.86 | <0.001 |
| Platelet | 1.00 | 0.99, 1.00 | 0.196 |
| RBC count | 0.88 | 0.76, 1.00 | 0.064 |
| Hemoglobin | 1.08 | 1.03, 1.13 | 0.002 |
| WBC count | 1.01 | 1.00, 1.01 | <0.001 |
| Serum creatinine | 0.91 | 0.87, 0.95 | <0.001 |
| Anion gap | 1.04 | 1.03, 1.05 | <0.001 |
| APTT | 1.00 | 1.00, 1.01 | <0.001 |
| INR | 1.11 | 1.08, 1.14 | <0.001 |
| Glucose | 1.00 | 1.00, 1.01 | 0.521 |
| CKD | 0.88 | 0.77, 1.02 | 0.083 |
| Diabetes | 0.89 | 0.79, 1.01 | 0.061 |
| Heart failure | 0.84 | 0.75, 0.95 | 0.005 |
| Hypertension | 0.84 | 0.75, 0.94 | 0.003 |
| Chronic liver disease | 1.17 | 1.01, 1.36 | 0.035 |
| COPD | 0.92 | 0.67, 1.26 | 0.587 |
| ARDS | 1.49 | 1.34, 1.67 | <0.001 |
| Cancer | 1.19 | 1.05, 1.34 | 0.005 |
| Coronary artery disease | 1.01 | 0.89, 1.15 | 0.847 |
| Stroke | 1.16 | 0.94, 1.43 | 0.159 |
| Sepsis | 1.16 | 1.03, 1.30 | 0.012 |
| MAP | 1.00 | 0.99, 1.01 | 0.240 |
| eGFR | 0.96 | 0.94, 0.98 | 0.001 |
| AKI stage | 1.12 | 0.71, 1.78 | 0.204 |
| SAPSII | 1.02 | 1.01, 1.03 | <0.001 |

Abbreviation: ﻿AKI, acute kidney injury; ﻿APTT, activated partial thromboplastin time; ﻿ARDS, acute respiratory distress syndrome; CI, confidence interval; COPD, chronic obstructive pulmonary disease; ﻿eGFR, estimated glomerular filtration rate; HR, hazard ratio; ﻿INR, international normalized ratio; MAP, mean arterial pressure; RBC, red blood cell; ﻿RRT, renal replacement therapy; ﻿SAPSII, Simplified Acute Physiology Score II; WBC, white blood cell.

**Table S3. Baseline characteristics of groups after propensity score matching analysis**

| Variables | Non-zinc group (n = 222) | Zinc group  (n = 222) | *P* | SMD |
| --- | --- | --- | --- | --- |
| Age | 66 ± 15 | 65 ± 14 | 0.250 | 0.124 |
| Sex, male, n (%) | 127 (57.2) | 125 (56.3) | 0.848 | 0.018 |
| Ethnicity, n (%) |  |  | 0.236 | 0.006 |
| White | 158 (71.2) | 153 (68.9) |  |  |
| Black | 20 (9.0) | 31 (14.0) |  |  |
| Other | 44 (19.8) | 38 (17.1) |  |  |
| Admission type, n (%) |  |  | 0.103 | 0.005 |
| Observation | 30 (13.5) | 36 (16.2) |  |  |
| Elective | 19 (8.6) | 7 (3.2) |  |  |
| Emergency | 133 (59.9) | 138 (62.2) |  |  |
| Urgent | 40 (18.0) | 41 (18.5) |  |  |
| Laboratory parameters |  |  |  |  |
| Platelet, K/μl | 188.5 ± 99.4 | 199.6 ± 127.5 | 0.308 | 0.087 |
| RBC count, K/μl | 3.2 ± 0.7 | 3.2 ± 0.7 | 0.748 | 0.029 |
| Hemoglobin, g/dl | 9.7 ± 2.0 | 9.6 ± 2.0 | 0.508 | 0.064 |
| WBC count, K/μl | 13.9 ± 7.3 | 13.4 ± 7.4 | 0.681 | 0.030 |
| Serum creatinine, mg/dl | 2.4 ± 2.6 | 2.2 ± 2.2 | 0.436 | 0.071 |
| Anion gap, mmol/L | 15.9 ± 5.3 | 15.8 ± 5.0 | 0.883 | 0.014 |
| APTT, seconds | 42.0 ± 25.8 | 42.4 ± 27.1 | 0.895 | 0.012 |
| INR | 1.7 ± 1.0 | 1.7 ± 1.1 | 0.706 | 0.035 |
| Glucose, mg/dl | 152.7 ± 73.0 | 148.4 ± 67.4 | 0.517 | 0.064 |
| Co-morbidities, n (%) |  |  |  |  |
| CKD | 62 (27.9) | 56 (25.2) | 0.519 | 0.010 |
| Diabetes | 67 (30.2) | 56 (25.2) | 0.243 | 0.114 |
| Heart failure | 59 (26.6) | 54 (24.3) | 0.586 | 0.053 |
| Hypertension | 48 (21.6) | 42 (18.9) | 0.479 | 0.069 |
| Chronic liver disease | 17 (7.7) | 17 (7.7) | 1.000 | 0.000 |
| COPD | 2 (0.9) | 2 (0.9) | 1.000 | 0.000 |
| ARDS | 45 (20.3) | 43 (19.4) | 0.812 | 0.023 |
| Cancer | 24 (10.8) | 19 (8.6) | 0.422 | 0.081 |
| Coronary artery disease | 45 (20.3) | 40 (18.0) | 0.546 | 0.059 |
| Stroke | 5 (2.3) | 5 (2.3) | 1.000 | 0.000 |
| Sepsis | 50 (22.5) | 39 (17.6) | 0.192 | 0.130 |
| MAP, mmHg | 80.9 ± 18.5 | 80.3 ± 18.3 | 0.734 | 0.032 |
| eGFR, ml/min/1.73 m^2^ | 52.4 ± 35.9 | 52.2 ± 36.1 | 0.974 | 0.003 |
| Mechanical ventilation, n (%) | 161 (72.5) | 148 (66.7) | 0.180 | 0.124 |
| Vasopressor use, n (%) | 100 (45.0) | 102 (45.9) | 0.849 | 0.018 |
| Anticoagulant use, n (%) | 32 (14.4) | 33 (14.5) | 0.893 | 0.000 |
| Antibiotic use, n (%) | 189 (85.1) | 183 (82.4) | 0.440 | 0.071 |
| AKI stage, n (%) |  |  | 0.699 | 0.006 |
| 1 | 178 (80.2) | 180 (81.1) |  |  |
| 2 | 8 (3.6) | 5 (2.3) |  |  |
| 3 | 36 (16.2) | 37 (16.7) |  |  |
| Scoring systems |  |  |  |  |
| SAPSII score | 44.7 ± 14.9 | 45.1 ± 15.3 | 0.792 | 0.025 |

Abbreviation: AKI, acute kidney injury; ﻿ARDS, acute respiratory distress syndrome; APTT, activated partial thromboplastin time; ﻿COPD, chronic obstructive pulmonary disease; eGFR, estimated glomerular filtration rate; ﻿INR, international normalized ratio; MAP, mean arterial pressure; RBC, red blood cell; SAPSII, Simplified Acute Physiology Score II; SMD, standardized mean difference; WBC, white blood cell.
